# You Were Better Than Expected–An Experimental Study to Examine Expectation Change in a Non-clinical Sample

**DOI:** 10.3389/fpsyg.2022.862946

**Published:** 2022-07-11

**Authors:** Rosa-Marie Groth, Winfried Rief

**Affiliations:** Department of Clinical Psychology and Psychotherapy, Philipps University of Marburg, Marburg, Germany

**Keywords:** expectation, expectation change, positive feedback, cognitive immunization, instruction, behavioral experiments

## Abstract

**Background:**

Reduced sensitivity to rewards as well as the tendency to maintain dysfunctional expectations despite expectation-disconfirming evidence (cognitive immunization) are considered core features of various mental disorders. It is therefore important for clinical research to have paradigms that are suitable to study these phenomena. We developed a new experimental paradigm to study explicit expectation change after prior expectation induction and violation. Its validity is tested by applying the paradigm to healthy individuals.

**Materials and Methods:**

In the main part of the study (experiment 1) we examined whether it is possible to change healthy individuals’ (Sample size 56) task-specific and generalized performance expectations through expectation-disconfirming experiences. We used a high-difficulty performance task to induce initially negative expectations regarding participants’ ability to successfully work on that unknown task. In the second part of the study, the difficulty of the test was lowered in one experimental condition, in order to disconfirm the negative expectations of the first part, while the other group continued with high test difficulty to confirm the negative expectations. We measured the participant’s explicit performance expectations before and after completing the tests. In experiment 2 (Sample size 57), we investigated the impact of different test instructions on expectation change. Using the same paradigm as in experiment 1, we added an “immunization-inhibiting” manipulation for one group and an “immunization-enhancing” manipulation for the other group.

**Results:**

In experiment 1, we were able to show that individuals changed their expectations according to variations of task difficulty. Adding instructions to manipulate cognitive immunization inhibited expectation change regardless of condition (experiment 2).

**Conclusion:**

Our approach allowed us to examine the effects of implicitly acquired performance expectations on explicit, verbalized expectation change. The new experimental paradigm used in this study is suitable to induce performance expectations, and to examine expectation-change among healthy individuals (experiment 1). Instructions to enhance or inhibit cognitive immunization processes both inhibited expectation change (experiment 2). The results are discussed within the context of current models of expectation change, cognitive immunization, and reward sensitivity.

## Introduction

Major depression disorder (MDD) is related to a reduced response to rewards (Henriques et al., 1994; Henriques and Davidson, 2000; Epstein et al., 2006; Foti and Hajcak, 2009; McCabe et al., 2009; Liu et al., 2011; Admon and Pizzagalli, 2015; Proudfit, 2015; Berry et al., 2019). However, appropriate responses to rewards (i.e., learning from rewards) are essential to change a negative interpretation bias, and to direct future behavior (Luft, 2014; Schulz, 2016). From a clinical (psychotherapeutic) point of view, it is crucial not only to understand how reward expectations establish, but also to investigate their modulation. For the successful therapy of mental disorders, we need knowledge about how to change negative expectations in order to support successful future behavior (Rief et al., 2015). Despite the importance of expectation change (in contrast to expectation development), the number of studies dealing with the shift of expectations is very limited. In this article, we aim to fill that gap by introducing a paradigm not only to manipulate the development, but also the change of expectations.

People who suffer from mental disorders do experience situations in which their expectations, some of which are specific to the disorder, are violated. Even a patient with MDD and a negative view of himself and the world experiences situations that contradict this negative view, e.g., that he succeeds in an exam he expected to fail, or that another person looks at him surprisingly friendly. Cognitive-behavioral psychotherapeutic interventions try to maximize the effect of these so-called “expectation violations” in order to revise the negative expectations (Doering et al., 2018). However, it could be shown that for patients with MDD, a violation of expectations does not automatically mean that the originally negative expectations are changed to more positive ones. The concept of “cognitive immunization” was introduced to explain cognitive strategies to maintain expectations despite contradicting experiences (Rief et al., 2015; Kube et al., 2017b). As a result, patients are likely to relapse into old behaviors (e.g., avoidance behavior) in the course of their illness, since the original expectation (e.g., to fail an exam) is maintained. To understand the process of how expectations are built and changed is therefore crucial to understand the disease itself and to design suitable therapeutic interventions (Rief and Joormann, 2019).

In this study, we refer to expectations as future-directed perceptions that relate to the occurrence or non-occurrence of a specific outcome (Laferton et al., 2017). In this regard, expectations are distinct from the term “belief” (Beck, 2011), which refers to a broader attitude toward, e.g., oneself, others and the future (e.g., “I am worthless”). Still, a psychological belief can lead to the formation of a future-directed expectation (e.g., “If I ask for help, they will say no”). We aimed to investigate the development, maintenance and shift of performance expectations. A performance expectation is the changeable belief that people form about their own current and future performance (Laferton et al., 2017; Kube et al., 2018a) and can be either specific (e.g., to succeed in a certain test) or general (e.g., to succeed in tests in general). Expectations of future performance has an impact on present behavior (e.g., avoiding performance situations versus tackling them) as well as on the self-concept (“loser” versus “winner”). Recent data suggests that contrary to healthy individuals, individuals with MDD tend to stick to their negative performance expectations despite expectation-disconfirming evidence (Kube et al., 2019a,b). The authors developed one of the first paradigms to systematically investigate the acquisition and change of expectations, the EXperimental Paradigm to investigate Expectation Change (EXPEC; Kube et al., 2018a).

These aforementioned studies are impressive proof of concepts to show that verbalized expectations can be built and changed in an experiment, but they do not provide sufficient information about how performance expectations arise and change under real-life conditions. With the present study, we want to extend these findings by investigating the acquisition and violation of performance expectations under more realistic conditions, hereby using a paradigm of probabilistic learning.

To develop a performance test to manipulate performance expectations, we applied four criteria: First, we had to make sure that the participants were not yet familiar with the test in order to avoid measuring the expectations from previous experiences. Second, participants should get direct feedback after each trial in order to slowly build up (part one of the experiment) and shift (part two of the experiment) expectations. Third, we were looking for a task where task difficulty and therefore the chance that a trial would be solved correctly could be varied constantly, and fourth, on the basis of the participant’s performance (instead of deceptive feedback, as in former experiments). Based on these four criteria, we opted for a modified time estimation task (Miltner et al., 1997; Holroyd and Krigolson, 2007), where participants have to respond in a specified time window (about one second after the sound of a tone). Afterward, participants receive feedback as to whether their answer was “on time” (correct, in the target time window) or not (incorrect).

The second aspect we wanted to focus on is the role of cognitions related to expectation-violating information. Studies show, that varying the cognitive appraisal of expectation-disconfirming experiences causes significant differences in expectation change (Kube et al., 2019a) in participants with MDD. This triggering of cognitive immunization through post-test instructions did not succeed in a non-clinical sample (Kube and Glombiewski, 2020), even though it was suggested based on the data of other studies (Mathews and Mackintosh, 2000). Given these contradicting study results we wanted to expand the results concerning cognitive-immunization processes by testing whether the induction of a depressive schema (Beck, 1963; Kube et al., 2018b) before the beginning of the test (e.g., “performing well in this test is arbitrary”) could induce a negative bias which could result in reduced expectation change. This is particularly relevant in clinical practice, since therapists should aim to initiate their behavioral experiments in a form that maximizes the change in expectations, and that prevents cognitive immunization.

After considering the preconditions listed above, the main aim of the present study was to test whether our paradigm is suitable for examining explicit expectation change (experiment 1).

Furthermore, we tested whether additional information in form of pre-test instructions that aim to modulate cognitive immunization strategies can alter expectation change (experiment 2).

We expected to find a change of expectations if prior induced negative expectations were violated unexpectedly and no change of expectation if prior induced negative expectations were confirmed (experiment 1). Furthermore, we expected to find larger expectation changes in individuals who received an immunization-preventing instruction prior to the violation of negative expectations than in those who received an immunization-enhancing instruction (experiment 2).

## Materials and Methods

Participants took part in a performance test, where previously acquired negative performance expectations were either violated or confirmed. Baseline (negative) performance expectations were built up continuously during the course of the first part of the test and explicitly assessed afterward. During the second half of the test participants either achieved an unchanged rate of positive feedback (expectation confirmation) or an unexpectedly higher rate of positive feedback (expectation violation), whereupon expectations were assed for the second time. Moreover, we tested whether different instructions (immunization-preventing or immunization-enhancing instructions) to the performance test could modulate expectation change.

**FIGURE 1:**
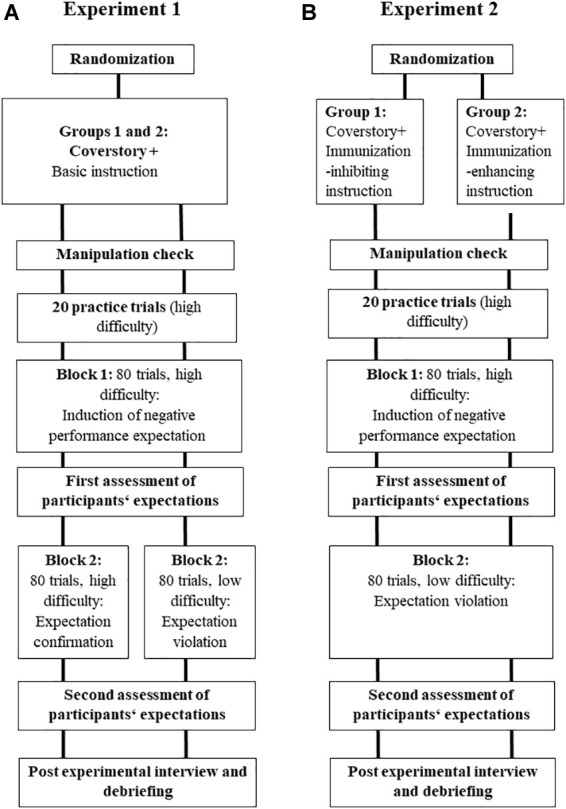
The basic procedure of the two experiments. Experiment 1 **(A)**: A cover story, a training block and a test block induced negative expectations regarding one’s ability to succeed in an unknown test. After the first test block, we assed participants’ expectations. The procedure continues with a second test block with either a high (group 1) or a low (group 2) difficulty to confirm (group 1) or disconfirm (group 2) the initial expectation. Afterward, we assessed participants’ expectations for the second time followed by a post-experimental interview and debriefing. Experiment 2 **(B)**: A cover story and additional immunization-inhibiting (group 1) or enhancing (group 2) instructions mark the beginning of the experiment. Afterward, we presented a training block as well as a test block induced negative expectations regarding one’s ability to succeed in an unknown test. After the first test block, we assed participants’ expectations. The procedure continues with a second test block low difficulty to disconfirm the initial expectation. Afterward, we assessed participants’ expectations for the second time followed by a post-experimental interview and debriefing.

The procedure of the study is illustrated in Figure 1.

### Ethics

The Local Ethics Committee of the Philipps University of Marburg approved the study (reference number 2019-56k). It was conducted in accordance with ethical standards as laid down in the Declaration of Helsinki and its amendments. All participants gave written informed consent and were treated in accordance with the ethical guidelines of the German Psychological Society.

### Participants

We determined the sample size via a *a priori* power analysis (expected *f* = 0.19; alpha = 0.05; power = 0.80). Consequently, the power analysis disclosed a sample size of 58 participants in each of the two experiments. The total sample consisted of 60 healthy participants in each experiment with a total sample size 120. They were randomly assigned to one of the two experiments. The inclusion criteria were an age of at least 18-years, sufficient German language skills, no current diagnosis of a mental disorder and no visual or hearing impairment. Participants were recruited via email lists. The experiment took up to 75 min and participants could gain course credit or money (10€) for their participation. As in the aforementioned paper, we excluded participants if they suspected the real purpose of the study in the post-experimental interview to avoid demand effects. Furthermore, we excluded participants with to low accuracy (<6% across all three blocks). The inclusion of this low accuracy would imply highly questionable data, suggesting that these participants did not understand the task and most importantly, the experimental manipulation (expectation violation) could not work. Based on these exclusion criteria, 113 participants remained in the final analysis with a sample size of 56 in experiment 1 and a sample size of 57 in experiment 2. The demographic data of these participants are listed in Table 1.

**TABLE 1:** Sociodemographic characteristics of the sample (*N* = 113).

	Experiment 1		Experiment 2	
	Expectation confirmation *N* = 28	Expectation violation *N* = 28	Expectation violation + Immunization-inhibiting instruction *N* = 29	Expectation violation + Immunization-enhancing instruction *N* = 28
Age in years, *M* (*SD*)	25.36 (8.59)	22.54 (7.13)	23.45 (3.61)	25.79 (11.55)
**Sex, *N* (%)**				
Female	21 (75.0)	19 (67.9)	19 (65.5)	18 (64.3)
Male	7 (25.0)	9 (32.1)	10 (34.5)	10 (35.7)
**Educational level, *N* (%)**				
No educational degree	0 (0)	1 (3.6)	0 (0)	0 (0)
Primary education	3 (10.8)	3 (10.8)	6 (20.4)	2 (7.2)
Secondary education	19 (67.9)	21 (75)	18 (62.1)	19 (67.9)
University degree	6 (21.4)	3 (10.7)	5 (17.2)	7 (25)

*M, Mean; SD, Standard deviation; N, Number.*

### Apparatus and Stimuli

The experiment was conducted in a laboratory room at the Philipps University of Marburg, Department of Clinical Psychology. All self-report measures were completed online via the survey platform SoSci Survey (Leiner, 2019). The performance task was performed using Presentation^®^ software Neurobehavioralsystems (2022).

### Instruction

Participants were given an information text concerning the purpose of the study. They were told that the study aimed to evaluate a test for its applicability for clinical diagnostic use, and further instructed that they were about to take a difficult, unknown test in order to test their cognitive flexibility (KonFlex). Participants were told that the test result could be an indicator of the successful performance of people in the private and professional area. This cover story aimed to highlight the importance of the test result and to induce neutral to negative performance expectations.

### Performance Task (KonFlex)

We used a time-estimation task, which required participants to estimate an interval of 1 s as accurately as possible (Miltner et al., 1997; Holroyd and Krigolson, 2007). The onset of each trial was indicated by an auditory cue (1000 Hz, duration 50 ms). Participants were required to press the ENTER key when they thought that 1 s had elapsed. After submitting their time estimation, participants received performance feedback informing them about the adequacy of their time estimation. This visual presentation of the feedback with the words “RICHTIG” (correct) or “FALSCH” (incorrect) for 1000 ms marked the ending of each trial. A tolerance time window around 1000 ms was used to indicate correct answers, see below. A 1400–1600-ms inter-trial interval was introduced before the next trial began, during which a white fixation cross on a black background was presented.

**FIGURE 2:**
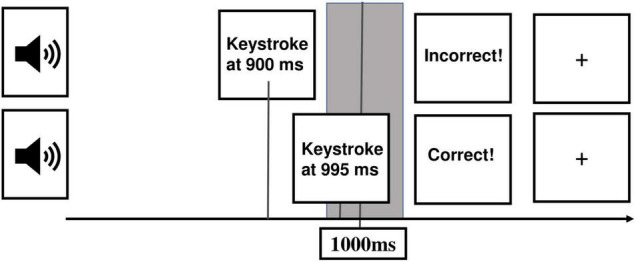
Illustration of a sequence of a trial. From the left part to the right part: The presentation of a sound (1000 Hz, duration 50 ms) marked the beginning of a trial. Afterward, participants estimate the duration of 1 s and give their answer via keystroke. Direct feedback appears depending on whether the button was pressed in the tolerance time window (gray field) around 1000 ms (correct) or not (incorrect). A fixation cross with an inter-trial interval of 1400–1600 ms is presented before the start of a new trial.

Before we introduced two experimental blocks, we presented a training block with 20 trials so that participants could get familiar with the task. These trials were excluded from further analyses. We presented 80 trials in each of the two experimental blocks. The first trial in each block was excluded from the analysis. In the previous paradigm (Kube et al., 2018a) participants received the expectation-confirming or disconfirming feedback just once and after an experimental block. In our study, participants received feedback after each trial so performance expectations could be built up continuously. The sequence of a trial is illustrated in Figure 2.

### Feedback, Task Difficulty, and Experimental Conditions

Participants were divided into two experiments with two experimental groups each. We varied the variables task difficulty (easy/hard) and instruction (immunization-inhibiting/immunization-enhancing).

#### Task Difficulty

Task difficulty was manipulated block-wise. In the first block, positive feedback was hard to achieve (high difficulty, induction of negative expectations). The time window’s length, in which an answer was classified as “correct,” was initially set from 990 to 1100 ms. By adjusting the time window trial wise, we assured that learning effects equally affected all participants. In the first block, we used the following criteria: in case of a false response, the window was widened by 3 ms; in case of an accurate response, the window was shortened by 12 ms, 6 ms at both boundaries. In the second block, participants either received expectation-confirming (experiment 1, group 1) or expectation-disconfirming (experiment 1, group 2) feedback. In the second experiment, participants received expectation-disconfirming feedback in the second block irrespective of condition. In the expectation-confirming condition, the criteria for adjusting the time window remained the same. In the expectation-disconfirming condition, the chance of positive feedback was higher because the algorithm changed to the following: in case of a false response, the window was widened by 12 ms; in case of an accurate response, the window was shortened by 3 ms. Subjects were not informed about this change in task difficulty.

#### Instruction

All participants (independent of experimental condition) were given the instruction described above. In experiment 2, we added information in order to inhibit or enhance cognitive immunization processes. In the “immunization-inhibiting” condition (experiment 2, group 1) we presented the instruction that the KonFlex has been shown to be very reliable and that the goal of the current study was to confirm its reliability in clinical use. Furthermore, participants were told that due to the high difficulty of the test, negative feedback at the beginning of the test was very likely. They were informed that an increase in positive feedback over the course of the test would represent outstanding skills. This instruction was supposed to sensitize for positive changes, and to make it less likely to engage in cognitive immunization strategies.

Group 2 (of experiment 2) received an “immunization-enhancing” manipulation, suggesting that the KonFlex has been shown to be highly controversial and that the goal of the current study was to refute its reliability. Participants in that condition were told that the feedback seems to be rather accidentally and therefore not meaningful. We predicted that this instruction would make it easier to engage in cognitive immunization strategies. Participants in experiment 1 received no additional information about the test. The other participants were given these immunization-enhancing or -inhibiting information before the beginning of the test in order to be able to draw conclusions about whether the initiation of a (behavioral) experiment influences the subsequent processing of the result.

### Measures

#### Socio-Demographics and Depressive Symptoms

Participants filled in a self-report questionnaire concerning their socio-demographic variables (age, nationality, mother tongue, education, and sex). Furthermore, we assessed depressive symptoms with the Beck’s Depression Inventory (BDI) II (Beck et al., 1996). The BDI II consists of 21 items, assessing depressive symptoms on a scale from 0 to 3. The internal consistency in our sample was α = 0.855.

#### Manipulation Check

We conducted a pre-experimental interview to ensure that participants fully understood the instruction. Participants could move on to the performance task, once they could correctly reproduce the relevant features (concerning the aim of the study, the task difficulty, and the significance of positive feedback) of the experiment.

After completing the first block, we asked the participants’ self-assessment of the test performance (“Please estimate what percentage of the tasks in the first block you solved correctly”) as well as their prediction for the second block (“Please estimate what percentage of the tasks in the second block you will solve correctly”). This way we could investigate whether our experimental manipulation of the instructions had an influence on expected performance development in the test. After the completion of the test, we asked the participants’ self-assessment of the test performance in the second block (“Please estimate what percentage of the tasks in the second block you solved correctly”).

#### Expectations

After completing the first test block, participants rated their task-specific and generalized performance expectations as well as five distractor items to not raise doubt about the purpose of the study, e.g., “I felt good during the test.” The item for task-specific expectation was “I will be successful in working on tasks from this test” and the one for generalized performance expectation was “I will be successful in working on unknown tasks in general.” The expectations were rated on a 6-point Likert Scale from (1) “I totally disagree” to (6) “I totally agree.” After completing the second block of the test, participants rated their task-specific expectations (“In the future, I will be successful to work on similar tasks like the ones from the test”) and generalized performance expectations (“I will be successful in working on unknown tasks in general in the future”) again as well as five distractor items, e.g., “I am happy with my result.”

#### Follow-Up Measures and Debriefing

We conducted a post-experiment interview in order to assess whether participants doubted the cover story. At the end, participants were fully informed about the actual purpose of the study and given the opportunity to ask questions.

### Analysis

For the KonFlex performance data, we calculated the mean accuracy for each participant and each block separately. We examined possible baseline differences between the four experimental conditions (expectation confirmation vs. expectation violation vs. immunization-inhibiting instruction vs. immunization-enhancing instruction) conducting a multivariate analysis of variance (MANOVA) on age, depressive symptoms, baseline expectations, and KonFlex performance (accuracy). We performed two chi-square tests of independence to examine the distribution of gender as well as education status across conditions. Changes in accuracy were analyzed using a 4 × 2 factorial ANOVA [4 × (Condition: expectation confirmation vs. expectation violation vs. immunization-inhibiting instruction vs. immunization-enhancing instruction) × 2 (time: block 1, block 2)].

For the manipulation check, we calculated a 4 (Condition: expectation confirmation vs. expectation violation vs. immunization-inhibiting instruction vs. immunization-enhancing instruction) × 2 (post-assessment block 1 vs. prediction block 2) ANOVA. We used contrasts to compare the two conditions of experiment 1 and the two conditions of experiment 2, as well as a contrast comparing experiment 1 to experiment 2.

For the main analysis, we conducted two 4 × 2 factorial ANOVAs for both task-specific and generalized expectations {4 (Condition: expectation confirmation vs. expectation violation vs. immunization-inhibiting instruction vs. immunization-enhancing instruction) × 2 [time: T0 (after block 1) vs. T1 (after block 2)]}. Also here, we used contrasts to compare the two conditions of experiment 1 and the two conditions of experiment 2, as well as a contrast comparing experiment 1 to experiment 2. Type-1 error levels were set at 5%. All analyses were conducted using IBM SPSS Statistics Version 22 (IBM Corp, 2013).

## Results

### Sample Characteristics

Due to the study design, there were no missing data. One participant did not understand the instruction due to insufficient German language skills and was therefore excluded from the analysis. As defined in the section “Materials and Methods,” another four participants were excluded because of low performance in the performance task (accuracy <6% across all blocks). An additional two participants were excluded because they confirmed the real purpose of the study in the post-experimental interview. Please note that on demand more participants expressed general doubts about the cover story, but did not mention the suspicion that the study could be about changing expectations. Therefore, we included 113 participants [with sample size 28 for the expectation confirming group, sample size 28 for the expectation violation group (sample size 56 for experiment 1), sample size 29 for the Expectation violation + Immunization-inhibiting instruction group, and sample size 28 for the Expectation violation + Immunization-enhancing instruction group (sample size 57 experiment 2)] in the final analysis. As indicated in Table 2, the absence of clinically relevant depressive symptoms for most participants was indicated by the mean BDI II score (*M* = 6.97, *SD* = 6.83) (Beck et al., 1996).

**TABLE 2:** Comparison of the four experimental conditions regarding baseline differences (*N* = 113).

	Experiment 1		Experiment 2	
	Expectation confirmation *N* = 28	Expectation violation *N* = 28	Expectation violation + Immunization-inhibiting instruction *N* = 29	Expectation violation + Immunization-enhancing instruction *N* = 28
	
	*M* (SD)	*M* (SD)	*M* (SD)	*M* (SD)
BDI	5.71 (5.57)	6.68 (5.91)	8.17 (7.12)	6.68 (7.38)
KonFlex accuracy Block 1	15.73 (7.16)	17.18 (5.53)	17.24 (6.43)	15.96 (5.96)
KonFlex accuracy Block 2	16.46 (7.81)	53.66 (9.70)	50.46 (9.01)	49.55 (12.49)
Baseline expectations: task specific	3.43 (1.35)	3.46 (1.07)	3.72 (1.03)	3.29 (1.24)
Baseline expectations: generalized	4.43 (0.79)	4.11 (1.1)	4.21 (1.08)	4.18 (0.95)
Expectations after task completion: task specific	3.07 (1.02)	3.82 (1.44)	3.31 (1.39)	3.04 (1.14)
Expectations after task completion: generalized	4.21 (1.00)	4.5 (0.88)	3.97 (1.32)	4.25 (1.18)

*M, Mean; SD, Standard deviation; N, Number; accuracy, percentage of correct answers.*

### Baseline Differences

Baseline differences were calculated between all four experimental groups to ensure comparability. Gender ratios were not significantly different between the four experimental groups χ^2^ (3) = 0.892, *p* = 0.827 which was also true for the educational level, χ^2^ (3) = 17.827, *p* = 0.467. As indicated by a MANOVA, participants from the four groups did not differ on age, *F*(3,109) = 0.998, *p* = 0.397; η^2^ = 0.027, BDI sum score, *F*(3,109) = 0.688, *p* = 0.561; η^2^ = 0.019, initial generalized expectations, *F*(3,109) = 0.552, *p* = 0.648; η^2^ = 0.015, initial task-specific expectations, *F*(3,109) = 0.688, *p* = 0.561; η^2^ = 0.019, and the first block of the KonFlex performance, *F*(3,109) = 0.449, *p* = 0.719; η^2^ = 0.012. As expected, results show a statistically significant difference between the experimental groups for the KonFlex performance in the second block, *F*(3,109) = 87.533, *p* < 0.001, η^2^ = 0.900. Bonferroni-adjusted *post hoc* analysis on accuracy data of the second block revealed significant differences between the expectation-confirmation group (experiment 1, group 1) and expectation-violation group (experiment 1, group 2), *p* < 0.001 (*M*_*Diff*_ = −37.21, 95%-CI [−44.31, −30.10]), immunization-inhibiting group (experiment 2, group 1), *p* < 0.001 (*M*_*Diff*_ = −34.00, 95%-CI [−41.05, −26.96]), and immunization-enhancing group (experiment 2, group 2), *p* < 0.001 (*M*_*Diff*_ = −33.09, 95%-CI [−40.20, −25.99]). Importantly, no differences were found between all other groups (*p* > 0.736), as indicated in Table 2. As expected, the Accuracy by Group mixed repeated measures ANOVA indicated a significant main effect of Time, *F*(1,109) = 1069.41; *p* < 0.001; η^2^ = 0.908 as well as a significant Time by Condition interaction *F*(3,109) = 112.41; *p* < 0.001; η^2^ = 0.756. Bonferroni-adjusted *post hoc* analysis on accuracy data of the repeated measures analysis revealed significant differences between the expectation-confirmation group (experiment 1, group 1) and expectation-violation group (experiment 1, group 2), *p* < 0.001 (*M*_*Diff*_ = −19.33, 95%-CI [−24.45, −14.20]), immunization-inhibiting group (experiment 2, group 1), *p* < 0.001 (*M*_*Diff*_ = −17.76, 95%-CI [−22.84, −12.67]), and immunization-enhancing group (experiment 2, group 2), *p* < 0.001 (*M*_*Diff*_ = −16.66, 95%-CI [−21.79, −11.53]). No baseline differences were found between all other groups (*p* > 0.989).

### Manipulation Check

The Time (post-assessment of block 1 vs. prediction of block 2) × Condition (expectation confirmation vs. expectation violation vs. immunization-inhibiting instruction vs. immunization-enhancing instruction) ANOVA including the post-assessment of block 1 (“Please estimate what percentage of the tasks in the first block you solved correctly”) as well as the prediction of block 2 (“Please estimate what percentage of the tasks in the second block you will solve correctly”) indicated a significant main effect of time, *F*(1,109) = 16.20; *p* < 0.001; η^2^ = 0.129, indicating a more optimistic expectation for the second experimental block independent of condition, as the Time × Condition interaction was non-significant *F*(3,109) = 1.290; *p* = 0.282; η^2^ = 0.034. The main effect of condition was non-significant (*p* = 0.282). With regard to experiment 1, there was no significant difference in the difference between post-assessment of block 1 and prediction of block 2 between the expectation violation and expectation conformation group, *F*(1,109) = 0.008; *p* = 0.930; η^2^ < 0.001. We observed a tendency in the direction of our hypothesis concerning the difference between the immunization-inhibiting instruction and the immunization-enhancing instruction group, *F*(1,109) = 3.670; *p* = 0.058; η^2^ = 0.033. There was no difference between the two experiments, *p* = 0.646. Table 3 shows the results of the manipulation check.

**TABLE 3:** Results of the manipulation check.

	Experiment 1		Experiment 2	
	Expectation confirmation *N* = 28	Expectation violation *N* = 28	Expectation violation + Immunization-inhibiting instruction *N* = 29	Expectation violation + Immunization-enhancing instruction *N* = 28
Post-assessment block 1, *M* (SD)	17.57 (16.08)	15.29 (10.37)	19.62 (18.59)	17.29 (16.78)
Prediction block 2, *M* (SD)	23.14 (21.58)	21.18 (13.05)	27.62 (21.74)	18.39 (20.21)
Post-assessment block 2, *M* (SD)	15.04 (10.00)	45.32 (19.01)	46.31 (20.21)	47.21 (21.88)

*M, Mean; SD, Standard deviation; post-assessment block 1, “Please estimate what percentage of the tasks in the first block you solved correctly”; Prediction block 2, “Please estimate what percentage of the tasks in the second block you will solve correctly”; post-assessment block 2, “Please estimate what percentage of the tasks in the second block you solved correctly.”*

### Main Analysis: Changes in Expectations

#### Change in Generalized Expectations

**FIGURE 3:**
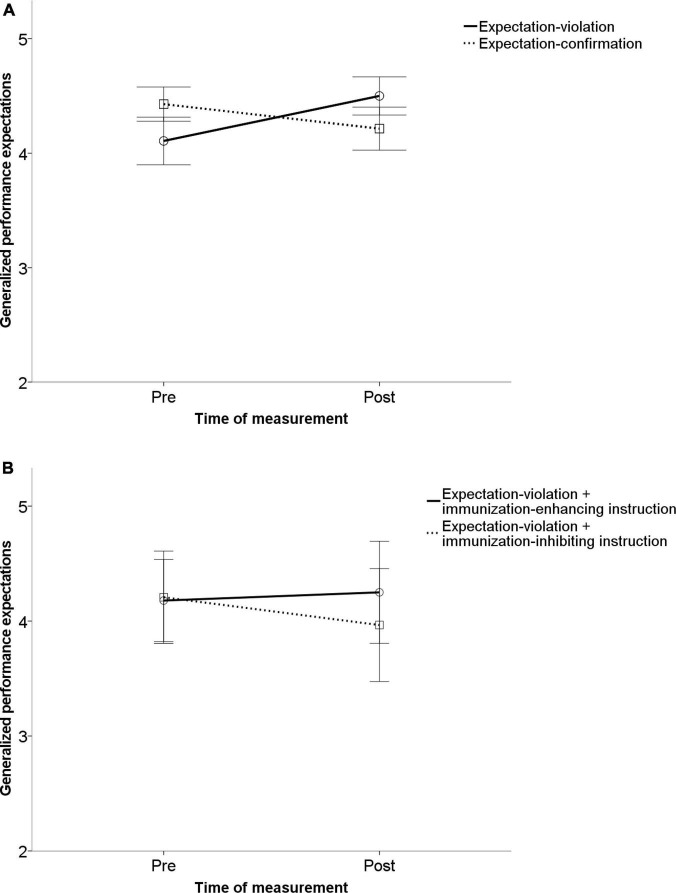
Illustration of the main results for generalized expectation change. Experiment 1 **(A)**: Results indicated that after receiving expectation-confirming performance feedback, participants did not change their generalized performance expectations significantly. In the expectation disconfirmation condition, participants significantly changed their generalized expectations. Experiment 2 **(B)**: No significant change of generalized expectations occurred after immunization-inhibiting or -enhancing instructions. The error bars represent one standard error.

With regard to generalized expectations (“I will be successful in working on unknown tasks in general in the future”) as the dependent variable, there was no significant main effect of time, *F*(1,109) = 0.001; *p* = 0.978; η^2^ < 0.001, generalized performance expectations were not significantly higher after the second block (*M* = 4.23, *SD* = 0.982) than after the first block (*M* = 4.23, *SD* = 0.110). Also, there was no significant main effect of condition, *F*(3,109) = 0.357; *p* = 0.784; η^2^ = 0.010. Change of generalized expectation interacted with the experimental condition (expectation confirmation vs. expectation violation vs. immunization-inhibiting instruction vs. immunization-enhancing instruction), *F*(3,109) = 3.531, *p* = 0.017, η^2^ = 0.089. With regard to experiment 1, there was a statistically significant difference in the change of generalized performance expectations between the expectation violation and expectation conformation group, *F*(1,109) = 7.347; *p* = 0.008; η^2^ = 0.063. Concerning experiment 2, we found no significant difference between the immunization-inhibiting instruction and the immunization-enhancing instruction group, *F*(1,109) = 1.985; *p* = 0.162; η^2^ = 0.018. There was no difference between the two experiments, *p* = 0.272. The results are visualized in Figures 3A,B.

#### Change in Task-Specific Expectations

**FIGURE 4:**
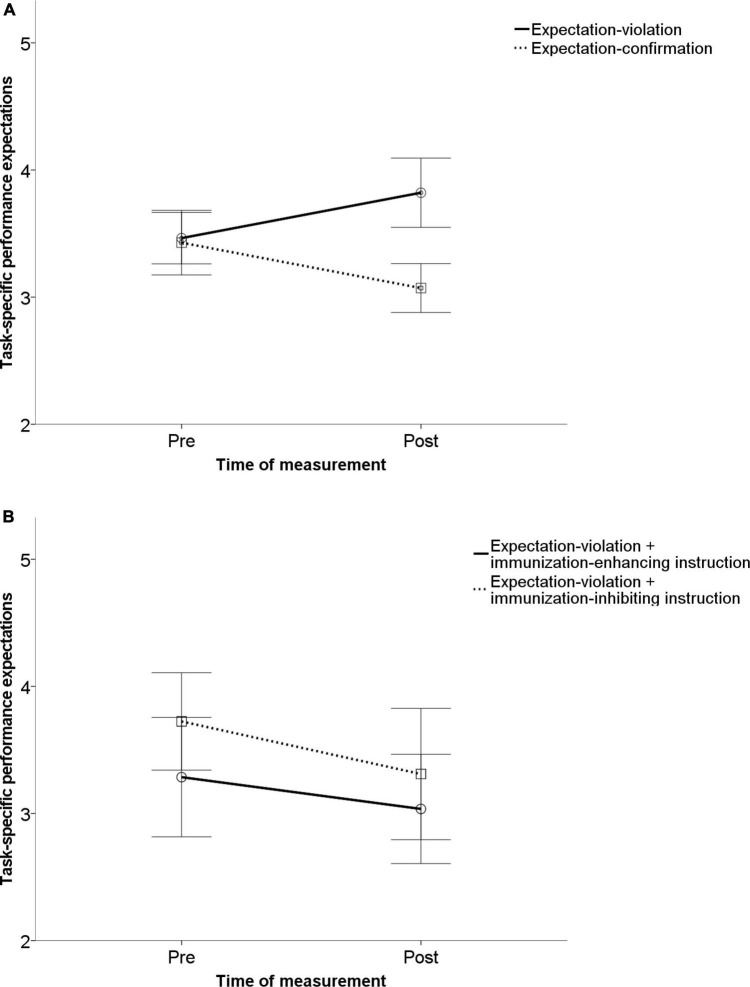
Illustration of the main results for specific expectation change. Experiment 1 **(A)**: Results indicated that after receiving expectation-confirming performance feedback, participants did not change their task-specific performance expectations significantly. In the expectation disconfirmation condition, participants significantly changed their task-specific expectations. Experiment 2 **(B)**: No significant change of task-specific expectations occurred after immunization-inhibiting or -enhancing instructions. The error bars represent one standard error.

Considering task-specific expectations (“I will be successful in working on tasks from this test”) as the dependent variable, there was no significant main effect of time (after block 1 vs. after block 2), *F*(1,109) = 1.85; *p* = 0.176; η^2^ = 0.017 or condition, *F*(3,109) = 1.335; *p* = 0.267 η^2^ = 0.035. The change of task-specific expectations did not interact with the experimental condition (expectation confirmation vs. expectation violation vs. immunization-inhibiting instruction vs. immunization-enhancing instruction), *F*(3,109) = 2.12, *p* = 0.102, η^2^ = 0.055. With regard to experiment 1, there was a difference in the change of task-specific performance expectations between the expectation violation and expectation conformation group, *F*(1,109) = 4.25; *p* = 0.042; η^2^ = 0.038, although it was non-significant at a Bonferroni-adjusted alpha level. Concerning experiment 2, we found no significant difference between the immunization-inhibiting instruction and the immunization-enhancing instruction group, *F*(1,109) = 0.227; *p* = 0.634; η^2^ = 0.002. There was no difference between the two experiments, *p* = 0.176. Figures 4A,B visualize these results.

## Discussion

In the present study, we introduced a novel paradigm to modulate performance expectations, and we evaluated whether it was suitable to determine explicit expectation change among healthy individuals. More precisely, the central question was, if participants who received unexpected positive feedback after a period of negative feedback changed their task-specific and generalized performance expectations, whereas patients in the expectation-confirmation condition did not. The most important changes we made to existing paradigms were (1) using a different, more implicit way to induce negative baseline expectations and (2) an implicit way to confirm or violate these baseline expectations. As hypothesized, we were able to replicate the main finding of the former paradigm (Kube et al., 2018a). Participants reported higher explicit generalized performance expectations after expectation-disconfirming positive feedback then after expectation-confirming negative feedback (experiment 1). The same tendency was observed for task-specific performance expectations. Therefore, our new-developed paradigm seems to be suitable to examine explicit expectation development and change with more implicit and individual induction of expectations. This finding is in accordance with recent studies showing that people in a non-clinical population can change their verbalized task-specific and generalized performance expectations after expectation-disconfirming experiences (Kube et al., 2018a,2019b).

Furthermore, we tested whether targeting a depressive schema in the immunization-enhancing condition (e.g., “performing well in this test is arbitrary” vs. “The test is very reliable and meaningful”) (Beck, 1963; Beck et al., 1979, 2016) before the beginning of the test could change the processing and interpreting of the new, unexpected positive information (experiment 2). The results of this experiment did not support the hypothesis that additional immunization-enhancing or -inhibiting instructions distinguishably alter explicit expectation change in healthy individuals. Surprisingly, in the main analysis of experiment 2, not only the expected interaction between the two conditions was absent, but also the main effect of time, indicating that participants from both experimental conditions did not significantly change their expectations after expectation-disconfirming feedback. Descriptive statistics revealed that in three of four pre to post expectations pairs, participants even reported a decrease of task-specific and generalized expectation (see Table 2). We found no evidence that participants in the second experiment (immunization enhancing vs. immunization inhibiting instruction) consciously perceived less positive feedback, as the manipulation check shows even higher self-assessed performance compared to the expectation-violation only condition (experiment 1, group 2) (see Table 3, Post-assessment block 2). Therefore, even though participants of experiment 2 reported to have achieved a much higher rate of positive feedback than previously expected, they did not change their expectations, while participants of the expectation-violation only condition (experiment 1, group 2) did. Ruling out a distortion of perception of positive feedback, especially the results of the immunization-inhibiting condition (experiment 2, group 1) appear to be surprising.

On the other hand, the analysis of the manipulation check confirmed that the experimental manipulation had an impact on the expected accuracy. Participants in the “immunization-enhancing” condition did not expect an increase of performance, while participants in the “immunization-inhibiting” condition did. The aforementioned result of the immunization-inhibiting condition (experiment 2, group 1) can be interpreted in line with studies claiming an optimism bias in a non-clinical population (Sharot et al., 2007; Sharot, 2011), whereas this effect was absent in the immunization-enhancing condition (experiment 2, group 2) and these participants rated their expected accuracy according to the “immunization-enhancing”-manipulation. Further, it should be considered that different processes could have led to similar results in both experimental conditions of experiment 2. However, given the relatively young research line of experimentally manipulating cognitive immunization, these results could also imply that under certain circumstance explicit expectation change is less stable and less coherent as previously expected. We believe that, especially due to publication bias (see for example Ferguson and Heene, 2012), it is important to report this null resort in order to provide as much data as possible for future research.

### Strength and Limitations

To start with, the assessment of task-specific and performance expectations with one item each is certainly not satisfying its complexity (Laferton et al., 2017). Notwithstanding we applied a manipulation check, whereby we overcame a limitation of previous studies and created a further indicator of explicit expectations by asking participants about the expected accuracy. Another limitation is the focus on performance expectation, even though MDD is also characterized by negative expectations in other areas (Backenstrass et al., 2006; Korn et al., 2014; Kube et al., 2017a,b). Furthermore, the analysis of individual performance estimates after block 1 (“Please estimate what percentage of the tasks in the first block you solved correctly”) and prediction of performance in block 2 (“Please estimate what percentage of the tasks in second block you will solve correctly”) of the test (manipulation check) revealed high variances. It can be assumed that the high variances made the detection of effects difficult. It may be that the subjects found it difficult to assess percentages in terms of correct answers and that it would have been more purposeful to ask for absolute numbers. With regard to the manipulation of the instructions, which in part did not cause the expected effects, one can speculate that the instructions themselves were too weak and possibly misleading. Further, more meaningful instructions would have to be tested in a further development of the present paradigm.

To move on to the strength of our study, it is important to emphasize that our new paradigm is highly flexible. Changes in task difficulty, number of trials, and instructions can, for example, be used to determine a minimum size of expectation violation that is needed to evoke explicit expectation change under certain circumstances. Moreover, our new paradigm enables researchers to include another aspect of expectation to future research, namely, their relation to reality, which is defined as the degree to how realistic an expectation is (Laferton et al., 2017). In our paradigm, this aspect can be captured by the manipulation check, where people are asked to rate their expected accuracy before the beginning of the test and self-evaluate their accuracy after completion.

Another strength of our paradigm is that the feedback is highly credible as it is directly linked to the participants’ behavior and we do not use deceptive feedback. Because despite the experimental manipulation in which an answer is classified as correct, participants still experience that they can influence which kind of feedback would occur. Note that this is especially true for the occurrence of incorrect feedback, as an extreme prolonging or shortening of the answer would always lead to negative feedback.

Most importantly, our paradigm provides an ecologically valid induction of performance expectations. While the EXPEC paradigm (Kube et al., 2018a) with its induction of expectations through an instruction and standardized, single feedback is essential as a proof-of-concept to approach the phenomenon of explicit expectation change, the reality is certainly more complex. So, without meeting the demands of studies on machine learning (Jordan and Mitchell, 2015; Yarkoni and Westfall, 2017), we used a more realistic form of expectation induction then the previous studies (Kube et al., 2018a,2019a; Kube and Glombiewski, 2020) by providing participants more “data” (in form of performance feedback) to build, confirm and violate their expectations. Consequently, we created our new paradigm in a way that enables researchers to explore biological correlates to cognitive immunization. The goal would be to bring forth a direct link between electrophysiological and neuroimaging deviations and the self-reported symptom of failed expectation change. An interesting first step would certainly be an implementation as an EEG experiment (for an introduction about the connection between reward expectation and event-related potentials see for example Cohen et al., 2007; Holroyd et al., 2008).

### Clinical Implications and Directions for Future Research

Due to the non-clinical population, the clinical implications of the current study are limited. Nevertheless, the paradigm is especially meaningful in a clinical context, as dysfunctional expectations are considered core symptoms of various mental disorders (Beck, 1963; Beck et al., 1979; Steele et al., 2007; Rief et al., 2015; Whitton et al., 2015). A centerpiece of cognitive behavior therapy is to enable the patient to approach situations with the aim of having expectation-disconfirming experiences that correct maladaptive schemata and thus to couple the person to the real environment (Beck et al., 1979). Although expectation-focused interventions differ from exposure therapy (Craske et al., 2014), behavioral experiments (Hamilton and Dobson, 2002), and interpersonal discrimination exercises (McCullough, 2003), such a focus can easily linked to these other techniques.

To succeed, these interventions need patients to correct their negative expectations after these expectation-disconfirming experiences. Therefore, the study of expectation change is highly relevant for the fields of clinical psychology. As a next step, studies should apply our paradigm to a clinical sample to evaluate to what extend the effects of “cognitive immunization” among people suffering from MDD (Kube et al., 2019a,b) can be replicated. Considering the research line of depressive realism (Ackermann and DeRubeis, 1991; Moore and Fresco, 2012), it could be revealing to use our manipulation check in order to examine whether people with MDD are better in self-evaluating their performance compared to a non-clinical population. Furthermore, given the results of the aforementioned study (Kube et al., 2019a), it can be assumed that the manipulations we used in experiment 2 has differentiated effects on people with MDD compared to healthy controls.

## Conclusion

Even though there is growing evidence that the reaction (in form of either explicit expectations or neural representations) to rewards is blunted in MDD and other mental disorders and different paradigms have been developed to measure this phenomenon, the extent to which these different results overlap, is yet unknown. Therefore, we modified an established paradigm (Kube et al., 2018a) to study expectation change and chose a more experience-based form of expectation induction. We were able to replicate the expectation-changing properties. Thus, the prerequisites to test the paradigm on a clinical sample are given. We were not able to confirm that triggering or preventing cognitive immunization processes had distinguishable effects in a healthy sample, but this effect needs further evaluation, e.g., in clinical samples and with more powerful instructions.

## Author’s Note

The authors assure that for all experiments, all measures, conditions, and terms for data exclusions are reported. The sample size was calculated based on the reported a priori power analysis. The code for a modified time estimation task is available upon request.

## Data Availability Statement

Due to privacy rules, the analyzed data are not openly available as conclusions about individual participants cannot be ruled out. Requests to access the datasets should be directed to rosa-marie.groth@uni-marburg.de.

## Ethics Statement

The studies involving human participants were reviewed and approved by the Ethics Committee of the Philipps University of Marburg. The participants provided their written informed consent to participate in this study.

## Author Contributions

R-MG was the principal investigator in terms of study design, implementation, and data collection and analysis, mainly involved in the development of the manuscript, and agreed to be responsible for all aspects of the work and to ensure that issues relating to the accuracy or integrity of any part of the work are adequately investigated and resolved. WR was substantially involved in the conception of the study, critically revision of the manuscript, and consents to the publication of the manuscript and agreed to be responsible for all aspects of the work and to ensure that issues relating to the accuracy or integrity of any part of the work are adequately investigated and resolved. Both authors contributed to the article and approved the submitted version.

## Conflict of Interest

The authors declare that the research was conducted in the absence of any commercial or financial relationships that could be construed as a potential conflict of interest.

## Publisher’s Note

All claims expressed in this article are solely those of the authors and do not necessarily represent those of their affiliated organizations, or those of the publisher, the editors and the reviewers. Any product that may be evaluated in this article, or claim that may be made by its manufacturer, is not guaranteed or endorsed by the publisher.
