# Corrigendum: You were better than expected—An experimental study to examine expectation change in a non-clinical sample

**DOI:** 10.3389/fpsyg.2022.1053536

**Published:** 2022-11-24

**Authors:** Rosa-Marie Groth, Winfried Rief

**Affiliations:** Department of Clinical Psychology and Psychotherapy, Philipps University of Marburg, Marburg, Germany

**Keywords:** expectation, expectation change, positive feedback, cognitive immunization, instruction, behavioral experiments

In the published article, there was an error in Figure 1, part B. The information for “Block 2” was displayed as “Block 2: 80 trials, high difficulty: Expectation confirmation.” The correct information is “Block 2: 80 trials, low difficulty: Expectation violation.” The corrected Figure 1 and its caption appear below. The caption and figure legend remain unchanged.

**Figure 1:**
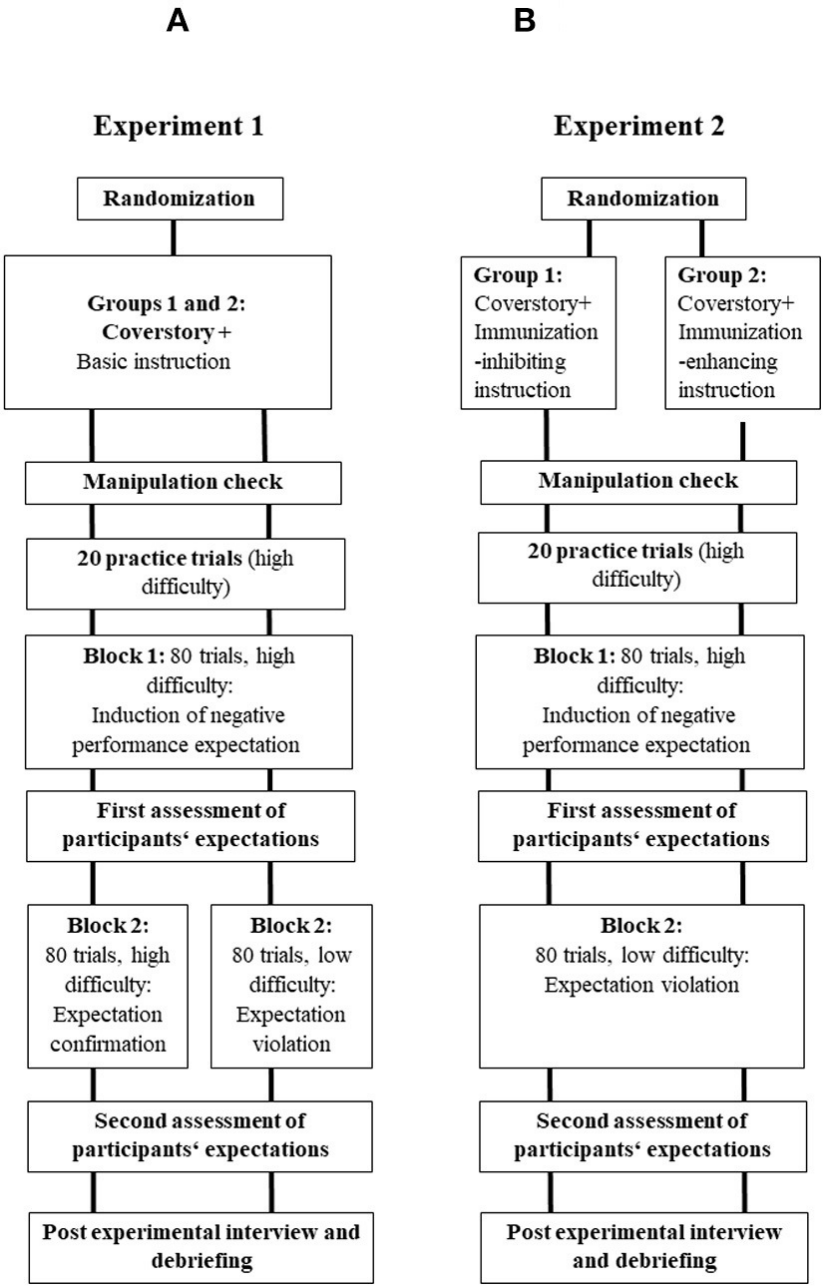
The basic procedure of the two experiments. Experiment 1 **(A)**: A cover story, a training block and a test block induced negative expectations regarding one's ability to succeed in an unknown test. After the first test block, we assed participants' expectations. The procedure continues with a second test block with either a high (group 1) or a low (group 2) difficulty to confirm (group 1) or disconfirm (group 2) the initial expectation. Afterward, we assessed participants' expectations for the second time followed by a post-experimental interview and debriefing. Experiment 2 **(B)**: A cover story and additional immunization-inhibiting (group 1) or enhancing (group 2) instructions mark the beginning of the experiment. Afterward, we presented a training block as well as a test block induced negative expectations regarding one's ability to succeed in an unknown test. After the first test block, we assed participants' expectations. The procedure continues with a second test block low difficulty to disconfirm the initial expectation. Afterward, we assessed participants' expectations for the second time followed by a post-experimental interview and debriefing.

The authors apologize for this error and state that this does not change the scientific conclusions of the article in any way. The original article has been updated.

## Publisher's note

All claims expressed in this article are solely those of the authors and do not necessarily represent those of their affiliated organizations, or those of the publisher, the editors and the reviewers. Any product that may be evaluated in this article, or claim that may be made by its manufacturer, is not guaranteed or endorsed by the publisher.

